# The Influence of the Digital Divide on Face Preferences in El Salvador: People without Internet Access Prefer More Feminine Men, More Masculine Women, and Women with Higher Adiposity

**DOI:** 10.1371/journal.pone.0100966

**Published:** 2014-07-09

**Authors:** Carlota Batres, David I. Perrett

**Affiliations:** School of Psychology and Neuroscience, University of St Andrews, St Andrews, Fife, United Kingdom; Brock University, Canada

## Abstract

Previous studies on face preferences have found that online and laboratory experiments yield similar results with samples from developed countries, where the majority of the population has internet access. No study has yet explored whether the same holds true in developing countries, where the majority of the population does not have internet access. This gap in the literature has become increasingly important given that several online studies are now using cross-country comparisons. We therefore sought to determine if an online sample is representative of the population in the developing country of El Salvador. In studies of Hispanic men and women aged 18–25, we tested facial masculinity and adiposity preferences by collecting data in person as well as online. Our results showed that there were no differences in preferences between people who reported having internet access, whether they were tested online or in person. This provides evidence that testing style does not bias preferences among the same population. On the other hand, our results showed multiple differences in preferences between people who reported having internet access and people who reported not having internet access. More specifically, we found that people without internet access preferred more feminine men, more masculine women, and women with higher adiposity than people with internet access. We also found that people without internet access had fewer resources (e.g. running water) than people with internet access, suggesting that harshness in the environment may be influencing face preferences. These findings suggest that online studies may provide a distorted perspective of the populations in developing countries.

## Introduction

Two traits that have been found to influence level of facial attractiveness are adiposity and sexual dimorphism. Facial adiposity refers to the perception of weight in faces [1] and has been found to serve as a cue to health [2]. One study found that Ugandan participants preferred heavier female figures than Greek and British participants [3]. Similarly, Swami and Tovée [4] found that, in Malaysia, urban participants found women with lower body mass indices to be more attractive than rural participants. Such findings have been suggested to arise due to differing optimal weights in different environments [4]. For instance, in environments with food shortages, heavier women may be better equipped to survive and reproduce [5] and therefore preferences for heavier women could be adaptive.

Sexual dimorphism refers to the differences between males and females. One study found that women in Jamaica preferred men with more masculine faces than women in the United Kingdom [6]. This finding has been attributed to the idea that health risks are higher in Jamaica than in the United Kingdom and therefore it would be beneficial for women in Jamaica to be more attracted to masculinity since there is some evidence that masculinity may signal health (e.g. [7], [8]). The evidence for the link between masculinity and health, however, is debatable [9], [10].

In order to further examine the relationship between masculinity preferences and health, DeBruine, Jones, Crawford, Welling, and Little [11] collected online data from 30 different countries. They found that masculinity preferences were negatively correlated with a computed health index of the country that the participants came from. This suggests that, in countries with poorer health, masculinity is considered more attractive because it is more important to have healthier offspring. On the other hand, Brooks et al. [12] proposed that national income inequality was a better predictor for masculinity preferences than the computed national health index. Brooks et al. suggested that in unequal societies, where women are less empowered and homicide rates are higher, masculinity preferences are stronger because masculinity signals dominance and male dominance is positively correlated with status [13], [14]. In response to Brooks et al.’s interpretation, DeBruine, Jones, Little, Crawford, and Welling [15] provided evidence that, among women from different states in the USA, health is a better predictor of masculinity preferences than both income inequality and homicide rates. This study thus showed that, even within the same country, sub-sectors of the population may be faced with different challenges and, as a result, exhibit differing levels of partner preferences.

Regardless of the interpretation used to explain masculinity preferences (i.e. [11] or [12]), it is important to consider the countries that were included in these online studies. Developed countries tend to have high levels of internet access. For example, 87% of the population in the United Kingdom has internet access [16]. Developing countries, in contrast, tend to have much lower levels of internet access. For instance, only 38% of the population in Mexico has internet access [16]. With such low levels of internet access in developing countries, it is unclear whether the online samples from these countries are fully representative of each country’s population.

The difference between people with internet access and people without internet access is commonly referred to as the digital divide [17], [18]. Past research has found that people with internet access tend to be wealthier and more educated than people without internet access [17]. It is important to understand the potential influence the digital divide has on partner preferences given that many experiments are now administered online [19], [20]. Previous studies on face preferences have found that online and laboratory experiments yield similar results with samples from developed countries (e.g. [19]). Yet no study has explored whether the samples used in online experiments are representative of the populations being examined in developing countries, where the digital divide is greatest. This gap in the literature has become increasingly important given that several online studies are now using cross-country comparisons [12], [11], [21]. Therefore, we sought to determine if an online sample is representative of the population in the developing country of El Salvador, where 26% of the population has internet access [16]. We also aimed to examine the extent of the digital divide in El Salvador by using questions intended to determine in what ways people with and without internet access differ.

### Hypotheses

We predicted that participants who reported having internet access would have similar face preferences, regardless of whether they were tested online or in person. We also predicted that participants without internet access would have different face preferences from participants with internet access. More specifically, we predicted that male masculinity would be considered more attractive by people without internet access than by people with internet access, since health risks [22] and homicide rates [23] are both higher in areas of El Salvador where internet is less accessible. Similarly, we predicted that adiposity would be considered more attractive by people without internet access than by people with internet access since health risks are higher [22] and reliability of access to food may be lower in areas without internet access.

## Methods

### Ethics Statement

Ethical approval was received from the University of St Andrews Ethics Board. Participants provided written consent after being presented with the information sheet and consent information.

### Study 1

#### Participants

69 men (M_age_ = 20.71 years, SD = 1.90) and 83 women (M_age_ = 20.46 years, SD = 2.09) aged 18–25 from El Salvador were recruited through word-of-mouth to complete the study in person. 31 men (M_age_ = 20.77 years, SD = 2.08; M_BMI_ = 25.67 kg/m^2^, SD = 4.73) and 40 women (M_age_ = 20.38 years, SD = 1.84; M_BMI_ = 23.29 kg/m^2^, SD = 4.04) reported having internet access in their home (internet in-person sample) while 38 men (M_age_ = 20.66 years, SD = 1.76; M_BMI_ = 21.15 kg/m^2^, SD = 2.07) and 43 women (M_age_ = 20.53 years, SD = 2.31; M_BMI_ = 22.47 kg/m^2^, SD = 3.07) reported not having internet access in their home (non-internet in-person sample). The majority of participants with internet access reported being from the state of San Salvador (83%) and the majority of participants without internet access reported being from the state of Ahuachapán (88%). It is important to note that San Salvador has higher health risks [22] and homicide rates [23] than Ahuachapán.

#### Materials

Face images of white men and women photographed facing forward, under constant camera and lighting conditions, with neutral expressions, no adornments, and closed mouths were selected from a commercially available library [24]. These images were delineated with 189 points using custom software [25] and aligned to a standard inter-pupillary distance [26]. Ten composite images (5 male and 5 female) were created (each averaging 3 original faces together) and masked to occlude clothes with a black oval around the head.

**Figure 1:**
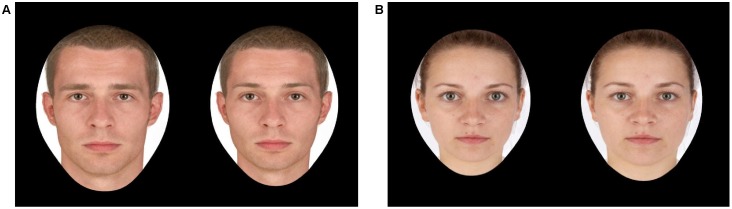
Example of Facial Stimuli. One of the male masculinity pairs (A), where the left face is masculinized in shape and the right face is feminized in shape. One of the female adiposity pairs (B), where the shape of the left face is decreased in BMI and the shape of the right face is increased in BMI.

The masculinity prototypes were generated by separately averaging female faces (M_age_ = 23.04 years, SD = 3.81) and male faces (M_age_ = 25.25 years, SD = 4.64) (for details see [27]). The male adiposity prototypes were generated by separately averaging male faces with a low body mass index (BMI) (M = 22.19 kg/m^2^, SD = 2.52; M_age_ = 25.10 years, SD = 3.96) and male faces with a high BMI (M = 26.47 kg/m^2^, SD = 3.27; M_age_ = 24.80 years, SD = 3.77). The female adiposity prototypes were generated by separately averaging female faces with a low BMI (M = 17.85 kg/m^2^, SD = 0.80; M_age_ = 22.70 years, SD = 3.56) and females faces with a high BMI (M = 24.06 kg/m^2^, SD = 6.34; M_age_ = 23.40 years, SD = 4.50) (for details see [28]). The prototypes were then used to create transforms with ±50% of the shape difference while holding texture and colour constant. This resulted in a total of 20 pairs of faces, where 10 pairs were of women and 10 pairs were of men. Among these 10 pairs, 5 pairs were made up of a feminized and masculinized face shape (see Figure 1A) and 5 pairs were made up of a low-BMI and a high-BMI face shape (see Figure 1B).

#### Procedure

Participants were tested individually and in person. Participants were tested without the use of computers given that participants without internet access were expected to be less familiar with computers than participants with internet access. Participants were given a stack of laminated sheets that consisted of 30 pairs of faces. The laminated sheets were blocked according to the sex of the face (15 faces were of men and 15 faces were of women). Each laminated sheet consisted of one pair of faces and which face appeared on the left/right was counterbalanced. Participants were asked to select which face from each pair they considered to be the most attractive. There was no time limit. The first 5 faces in each block consisted of faces that differed in perceived height and served to familiarize the participants with the task of selecting which face they considered to be the most attractive. The remaining 10 pairs in each block consisted of the faces that differed in the traits of interest (i.e. masculinity and adiposity).

The participants then completed a questionnaire that was administered verbally in Spanish which requested the participant’s sex, age, which state they were from, whether they had internet access in their home, and several other questions intended to determine in what ways people with and without internet access differ: whether they graduated from high school, whether they were attending or had graduated from university, whether they had children, whether they have a television in their home, whether they were born in a hospital, whether they have running water in their home, and how many times they have been to other countries. The questionnaire was administered verbally given that some of the participants were expected to be unable to read and write. Lastly, their height and weight were measured. Each participant was given 5 US dollars upon completion of the experiment.

### Study 2

#### Participants

17 men (M_age_ = 20.71 years, SD = 2.02) and 28 women (M_age_ = 20.43 years, SD = 1.57) aged 18–25 from El Salvador were recruited online. Everyone in this sample reported having access to the internet in their home (internet online sample). The majority of participants reported being from the state of San Salvador (82%).

#### Procedure

The procedure was identical to that in Study 1 except that it was conducted online and therefore all questions were administered in a written format in Spanish. The pairs of faces were presented in the same manner as they were in Study 1 except that they were presented online and participants clicked on the face they considered the most attractive. Participants were not paid for their participation.

## Results

**Figure 2:**
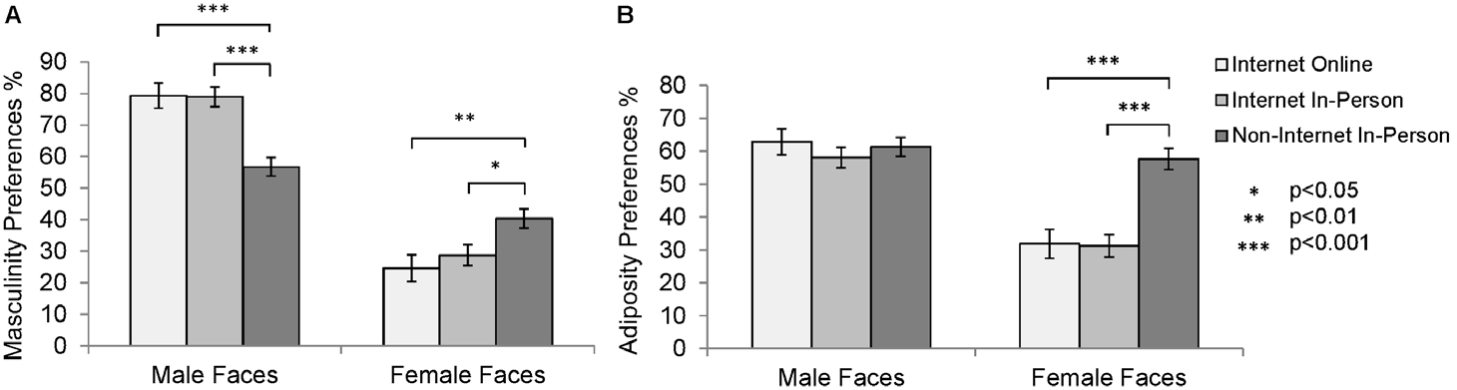
Masculinity and Adiposity Preferences. Comparisons of (A) masculinity preferences and (B) adiposity preferences between the internet online sample (light grey bars), the internet in-person sample (medium grey bars), and the non-internet in-person sample (dark grey bars). Preferences refer to the marginal mean percentage of faces high on the trait selected across the pairs.

Masculinity and adiposity preferences were calculated by taking the percentage of faces high on the trait selected across the pairs. One sample t-tests revealed that the faces selected for both traits in both sexes were significantly different to chance in all three samples (p<0.032 for all comparisons). Age was not significantly different between the samples (F(2,194) = 0.016, p = 0.984). Data were analysed using ANCOVAs (fixed factors: sample (3 levels: internet in-person, non-internet in-person, internet online) and sex of participant (2 levels) (see Table S1 for a summary of the results). The ANCOVAs revealed no significant effects of sex of participant (p>0.201 for all analyses) as well as no significant interaction between sample and sex (p>0.383 for all analyses). The ANCOVAs did reveal a significant effect of sample for all the analyses except adiposity preferences in male faces. Post-hoc tests with a Bonferroni correction were conducted for the preferences where sample had a significant effect (see Figure 2).

The ANCOVA for masculinity preferences in male faces revealed a significant effect of sample (F(2,191) = 16.872, p<0.001, η_p_^2^ = 0.150), where the non-internet in-person sample preferred more feminine male faces than both the internet in-person sample (p<0.001) and the internet online sample (p<0.001). The ANCOVA for masculinity preferences in female faces revealed a significant effect of sample (F(2,191) = 5.671, p<0.01, η_p_^2^ = 0.056), where the non-internet in-person sample preferred more masculine female faces than the internet in-person sample (p<0.05) and the internet online sample (p<0.01). The ANCOVA for adiposity preferences in male faces revealed no significant effect of sample (F(2,191) = 0.503, p = 0.605, η_p_^2^ = 0.005). The ANCOVA for adiposity preferences in female faces revealed a significant effect of sample (F(2,191) = 19.553, p<0.001, η_p_^2^ = 0.170), where the non-internet in-person sample preferred faces with higher adiposity than both the internet in-person sample (p<0.001) and the internet online sample (p<0.001). Post-hoc tests revealed no significant differences between the internet in-person sample and the internet online sample (p>0.9 for all comparisons).

The non-internet in-person sample reported having been to other countries less than both the internet in-person (t(150) = 17.142, p<0.001) and the internet online samples (t(124) = 14.950, p<0.001). Compared to the internet in-person sample and the internet online sample, the non-internet in-person sample was also less likely to have a television (χ^2^(1) = 17.897, p<0.001; χ^2^(1) = 11.667, p<0.01), more likely to have children (χ^2^(1) = 26.227, p<0.001; χ^2^(1) = 16.975, p<0.001), less likely to have graduated from high school (χ^2^(1) = 84.525, p<0.001; χ^2^(1) = 61.642, p<0.001), less likely to have attended or graduated from university (χ^2^(1) = 144.185, p<0.001; χ^2^(1) = 117.660, p<0.001), less likely to have been born in a hospital (χ^2^(1) = 38.639, p<0.001; χ^2^(1) = 28.441, p<0.001), and less likely to have running water in their home (χ^2^(1) = 24.981, p<0.001; χ^2^(1) = 16.471, p<0.001). The internet in-person and internet online samples did not differ in any of the above (p>0.426 for all comparisons) (see Table S2 for the descriptive statistics for the three samples).

## Discussion

Our results showed that there were no differences in preferences between people from El Salvador who reported having internet access, whether they were tested online or in person. This provides evidence that testing style does not bias preferences among the same population. On the other hand, our results showed multiple differences in preferences between people from El Salvador who reported having internet access and people from El Salvador who reported not having internet access. This suggests that, unlike samples from studies conducted online with participants from developed countries (e.g. [15]), samples from studies conducted online with participants from developing countries may not be fully representative of the populations (e.g. [12], [11], [21]). Future research needs to take this into account when using online samples from countries where a substantial portion of the population does not have internet access. This applies not only to face preference research but to all studies that use online testing in developing countries (e.g. [29]).

Our data provide evidence that, even within a small country, sub-sectors of the population have different preferences. We found that adiposity preferences in female faces were higher among people without internet access than people with internet access. This finding is consistent with previous literature that has found that heavier figures are considered more attractive in poorer and rural areas [3], [4].

Contrary to our expectations, we found that masculinity in male faces was considered more attractive by people with internet access than by people without internet access. Past research has suggested that risks to health from disease [11] or violence [12] may be responsible for differing levels of masculinity preferences in male faces. Neither interpretation holds for face preferences within El Salvador since we found that participants without internet access prefer more feminine male faces even though health risks [22] and homicide rates [23] are both higher in areas of El Salvador where internet is less accessible. Further research is needed in order to determine what is driving these differing face preferences within sub-sections of the population.

One possibility is that media exposure is driving both sexual dimorphism and adiposity preferences. Several studies have found that the media promotes certain beauty ideals, such as masculinity in men, and femininity and low body weight in women [30], [31]. People who have internet access experience greater exposure to the media through online advertisements and websites and are therefore likely to be more exposed to faces with accentuated masculinity and femininity as well as female faces with lower adiposity.

We also found that participants with internet access were more likely to have a television in their home, which exposes them even further to the media through commercials, television shows, and movies. For example, starring movie roles are more likely to be played by women with low body mass indices [31]. Exposure to such beauty ideals has been found to impact behaviour and preferences. For instance, one study found that adolescent Fijian girls became more interested in weight loss after television was introduced in their town [32]. Thus, media exposure may explain our findings of preferences for higher masculinity in male faces and higher femininity and lower adiposity in female faces among people with internet access in El Salvador. Under the media exposure interpretation, however, it remains unclear why past research has found that online participants from developing countries prefer more masculine male faces than online participants from developed countries [11], since people from developing countries tend to have lower levels of media exposure than people from developed countries [33].

A second explanation for our findings is that the level of harshness in the environment may be influencing face preferences. Our data provide evidence that people without internet access face a harsher environment than people with internet access. For example, we found that people without internet access are less likely to have access to running water in their home than people with internet access. One study found that women prefer less masculine men and men prefer more masculine women for long-term relationships when they are asked to imagine themselves in harsh circumstances [20]. Therefore, increased levels of environmental harshness could explain our findings of preferences for masculine women and feminine men among people without internet access.

The environmental harshness explanation could also explain our adiposity findings. Past research suggests that BMI preferences may reflect differing optimal weights in different environments [4]. For instance, heavier women are better equipped to survive in periods of famine [5] and therefore may be found more attractive in environments with food shortages. Although BMI and weight were higher among people with internet access, preferences for adiposity were higher among people without internet access. This suggests that, although higher levels of weight are considered more attractive in the non-internet population, it may be harder to achieve high levels of weight in such a harsh environment.

Although the environmental harshness explanation is consistent with our findings, further research is needed in order to identify what forms of hardship are most influential on preferences. For instance, Lee and Zietsch [34] found that when women are primed with pathogen prevalence they prefer good-gene traits, such as ‘muscularity’, but when they are primed with resource scarcity they prefer good-dad traits, such as ‘nurturing’. In an environment like El Salvador, where both pathogen prevalence and resource scarcity are real threats, it remains to be determined which form of hardship is more influential on preferences. It may be possible that, among people with internet access in developing countries, pathogen prevalence is more influential since they face less resource scarcity. This would explain why past studies have found that masculinity preferences are negatively correlated with country-level health indices in online samples [11]. On the other hand, people without internet access face both pathogen prevalence as well as resource scarcity. Using Lee and Zietsch’s [34] findings, our studies provide some preliminary evidence that resource scarcity may be more influential than pathogen prevalence in environments with both threats since our non-internet sample preferred more feminine men. In order to confirm this preliminary analysis, more sensitive questions that measure resource scarcity would need to be used in future studies.

In addition to the differences in access to television and running water, we also found that people without internet access have been to other countries fewer times, have children earlier, are less educated, and are less likely to have been born in a hospital than people with internet access. These differences suggest that people with internet access have very different lifestyles from people without internet access, which provides further evidence of a digital divide [17], [18]. Our findings show that the digital divide does influence face preferences and this relationship needs to be taken into consideration in future experiments in order to accurately measure the preferences of people from developing countries.

One limitation from our experiment is that, unlike our in-person samples, our online sample was neither compensated nor supervised and participants might therefore be less motivated to take the experiment seriously. Past studies, however, have found that participants who are uncompensated and unsupervised yield results that are comparable in quality to participants who are compensated and supervised [35]. Our study was also limited in that our experiment consisted of only 5 trials per condition, it only used faces of white men and women, and our participants came from only one country. It would be beneficial to examine if any differences in face preferences arise from using faces of another ethnicity versus faces of own ethnicity. Additionally, although all Salvadorians fall under the ethnicity of Hispanic, there are differences within this ethnicity that may reflect cultural and genetic heritage and might influence preferences. It would therefore be beneficial for other studies to examine the influence of the digital divide within other developing countries. While it is clear from past studies that preferences for facial characteristics differ across populations (e.g. [11]), there are a number of factors that can contribute to these differences (e.g. health [15], violence [12], societal-level measures of development [21], income inequality [12], ecological conditions [36], media [32]). In order to gain a better understanding of these influences, more studies that compare sub-sectors of the same geographical population (e.g. [37], [36]) need to be undertaken.

## Supporting Information

Table S1 Summary of ANCOVA results.
(XLSX)

Table S2 Descriptive statistics for the three samples.
(XLSX)
