# Correction: The Influence of the Digital Divide on Face Preferences in El Salvador: People without Internet Access Prefer More Feminine Men, More Masculine Women, and Women with Higher Adiposity

**DOI:** 10.1371/journal.pone.0155279

**Published:** 2016-05-04

**Authors:** Carlota Batres, David I. Perrett

There are errors in the published article. The authors incorrectly note that homicide rates are lower in San Salvador than in Ahuachapán. The authors have found that homicide rates are actually greater in San Salvador than Ahuachapán.

In the Introduction, the Hypotheses paragraph should read as follows: “We predicted that if health risks are a better predictor of masculinity preferences as suggested by Debruine et al. [11], then male masculinity would be considered more attractive by people without internet access than by people with internet access, since health risks [22] are higher in areas of El Salvador where internet is less accessible. If, however, homicide rates and income inequality are better predictors of masculinity preferences as suggested by Brooks et al. [12], then male masculinity would be considered more attractive by people with internet access than by people without internet access, since homicide rates and income inequality [23] are higher in areas of El Salvador where internet is more accessible.”

In the Methods, the last sentence of the Participants paragraph for Study 1 should read as follows: “It is important to note that San Salvador has lower health risks [22] but higher homicide rates [23] than Ahuachapán.”

The third paragraph of the Discussion is incorrect. The correct paragraph is: “We also found that masculinity in male faces was considered more attractive by people with internet access than by people without internet access. Past research has suggested that risks to health from disease [11] or violence [12] may be responsible for differing levels of masculinity preferences in male faces. In our sample, participants without internet access face higher health risks [22] but lower homicide rates [23]. Our results, thus, support Brooks et al.’s [12] interpretation that homicides rates and income inequality are better predictors of masculinity preferences than health risks since we found that male masculinity was considered more attractive by people with internet access than by people without internet access.”

Reference 22 is incorrect. The correct reference is: Anuario Estadistico (2009) Ministerio de Economia. Gobierno de El Salvador.
